# Novel technique in difficult percutaneous tracheostomy

**DOI:** 10.4103/1658-354X.76466

**Published:** 2011

**Authors:** Babita Gupta, Manpreet Kaur, Nita D’souza, Chandni Sinha

**Affiliations:** *Department of Anaesthesia, All India Institute of Medical Sciences, J.P.N.A Trauma Centre, New Delhi, India*

Sir,

Percutaneous tracheostomy has become an appropriate alternative to conventional surgical tracheostomy and has gained worldwide acceptance as a bedside procedure.[1] Despite the best efforts, complications like false passage are known to occur with percutaneous tracheostomy. Forceful attempts through false passage introduce the potential for significant hemorrhage and disruption of the tract, further aggravating the situation.

We describe a technique using a standard nasogastric tube (NGT) that allows precise and safe placement of tracheostomy tubes in patients under emergency situation of false passage. This method eliminates the need for specialized equipment and skills like fiberoptic which is usually not available at bedside where percutaneous tracheostomy actually takes place.

A 22-year-old male patient operated for right fronto-temporo-parietal subdural hematoma required tracheostomy. Attempt to percutaneous tracheostomy using the Ciaglia’s Blue Rhino technique created a false passage. Repeated attempts to negotiate tracheostomy tube over guide wire failed, leading to airway emergency scenario. We devised a novel technique to overcome the false passage.

**Figure 1:**
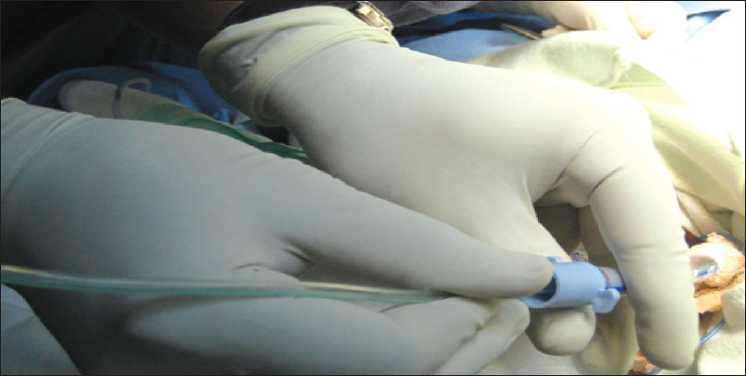
Insertion of Ryle’s tube through the ETT

**Figure 2:**
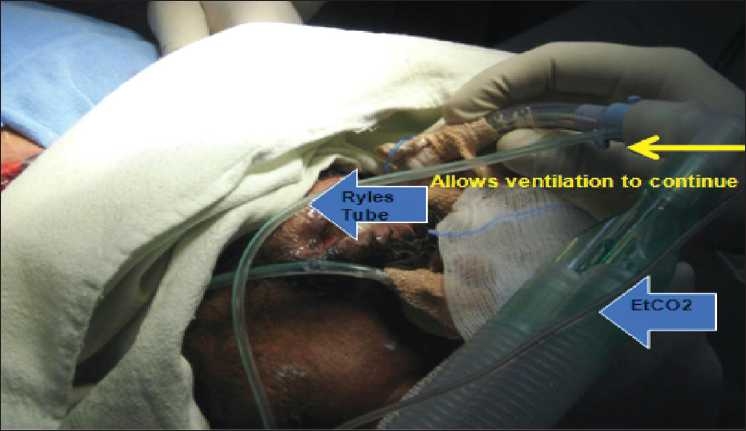
Ryle’s tube inside the ETT and ventillation being continued by attaching the ETT to ventilator tubing

**Figure 3:**
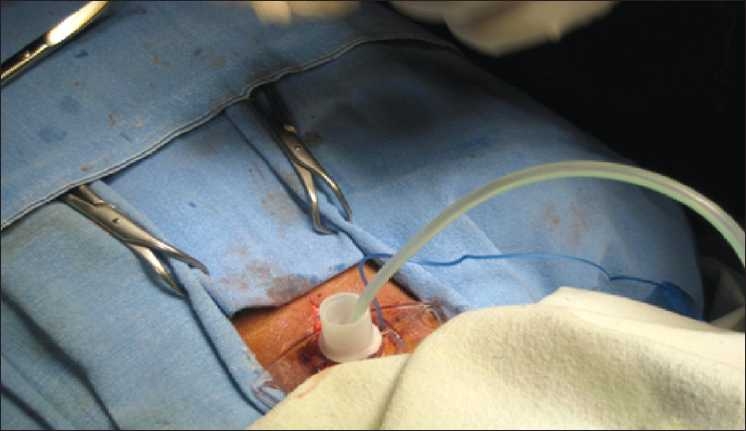
Railroading tracheostomy tube over the Ryle’s tube

An endotracheal tube (ETT) which had been withdrawn upto glottis was pushed caudally so that it occupied the distal end of the trachea, sealing off the rent created by dilation of the tracheostomy tract. A Ryle’s tube with its tip cut was inserted through the ETT already *in situ* [Figure 1]. The ETT served as a guide for the Ryle’s tube into the trachea. The ETT was again withdrawn upto the glottis so that the Ryle’s tube could be visualized through the rent in the trachea. Oral end of the Ryle’s tube was taken out through the tracheal stoma with distal end in distal end of trachea [Figure 2]. The NGT serves as a guidewire for the tracheostomy tube, which is then advanced gently over the NGT and into the trachea. After the tracheostomy tube is in place, the NGT is removed. Oxygenation and ventilation was continued during the whole procedure [Figure 3]. Initially when the NGT passed through the ETT, its proximal end passed under the ventilator attachment and later when NGT oral end was out of tracheal stoma supplemental oxygen was hooked into the NGT to help ameliorate hypoxia. The fiberoptic endoscope is an excellent method for the proper placement of tracheostomy tubes[2] and prevention of false passage. Its use, however, requires some expertise and experience that will not be in the armamentarium of all and besides its availability at the bedside is limited. So we devised an easy and readily available technique to overcome the airway emergency situation. With routine, scheduled tracheostomy tube changes, the situation may be similar. Alternative “guidewires-red rubber catheter, suction catheter” techniques are less beneficial because they are too flexible and may not provide an adequate guide for the tube into the trachea.[3] Limitation to this technique is that the inner tube has to be removed for railroading of tracheostomy tube over Ryle’s tube. False passage can be a challenging and a frightening procedure for the physician. In a difficult tracheostomy scenario or during creation of a false passage, tracheostomy tube can be railroaded over the Ryle’s tube.
